# Lectotypification of names of Himalayan Brassicaceae taxa currently placed in the genus *Cardamine*

**DOI:** 10.3897/phytokeys.50.5080

**Published:** 2015-05-13

**Authors:** Karol Marhold, Matúš Kempa, Ihsan A. Al-Shehbaz

**Affiliations:** 1 Department of Botany, Faculty of Science, Charles University, Benátská 2, CZ-128 01 Praha 2, Czech Republic; 2 Institute of Botany, Slovak Academy of Sciences, Dúbravská cesta 9, SK-845 23 Bratislava, Slovakia; 3 Missouri Botanical Garden, P.O. Box 299, St. Louis, Missouri, 63166-0299, U.S.A.

**Keywords:** *Cardamine*, Himalaya, nomenclature, stable identifiers for specimens, taxonomy, typification

## Abstract

Lectotypes of twenty-eight names of taxa currently recognized or synonymized in *Cardamine* are designated as part of the work on the account of the genus for the Pan-Himalayan Flora. Among them, the previous first-step lectotypification of the name *Cardamine calthifolia* is finalized. In cases when specimen images are available online, stable identifiers for specimens, other permanent links, or links via JSTOR Global Plants are provided.

## Introduction

During the work by the last author on the Brassicaceae (Cruciferae) account for the Pan-Himalayan Flora (PHF), it became evident that the majority of accepted names and their synonyms in that flora require lectotypification. The present paper focuses on the lectotypification of names of taxa currently placed in *Cardamine* L., a genus with 43 species in the PHF.

## Materials and methods

Herbarium specimens, especially types and authentic collections, deposited at B, BM, E, F, G, GH, K, LE, MO, NAS, NY, P, US, W, and WU were examined during the past two decades. In cases when specimen images are available online, stable identifiers for specimens (Hyam et al. 2012, Güntsch and Hagedorn 2013, Hagedorn et al. 2013; in the case of specimens from herbaria B, E, K), other permanent links (herbaria W, WU, see JACQ Consortium 2004 onwards; F, MO, P) or links via JSTOR Global Plants (https://plants.jstor.org/; herbaria BM, GH, NY, US) are provided. We suggest that the practice of providing stable indentifiers or other kind of permanent links to images of herbarium specimens should be adopted as standard one for lectotypification papers. It will make eventual registration or evidence of designated types much easier. The bibliographical citations in the original publications and databases such IPNI (The International Plant Names Index; http://ipni.org/), Tropicos (http://www.tropicos.org/), and The Plant List (http://www.theplantlist.org/) were also checked.

In lectotypifying names of taxa, we strictly followed the International Code of Nomenclature for algae, fungi, and plants (McNeill et al. 2012) and the recommendations recently presented by McNeill (2014). In cases where a single specimen is known that was used by the describing author and no duplicates were found or were not expected to exist, that particular specimens is considered a holotype, provided that it meets the criteria given in the publications above. In cases where one or more duplicates of the type collection exist (or possibly or likely existed) or more than one syntype was cited in the original publication, we first checked the material housed in the institution where the author(s) of the name worked. For example, for taxa described by Adrien René Franchet, Joseph Dalton Hooker, and Otto Eugen Schulz, we first checked the material deposited in the herbaria P, K, and B, respectively. The best of all duplicates that do not contradict the protologue or the guidelines in the *Code* is designated as the lectotype. If the home institution of the author of the name does not have any original material and no herbarium was cited, we put emphasis on the material annotated by that name author. If none of the above cases applies, the designation was based on the best representative material.

Entries are arranged alphabetically by their basionyms, and names in boldface are those of currently accepted names of taxa. Bibliographic citations are given for all names and only examined and designated lectotypes and their duplicates are listed. Barcode numbers of lectotypes and isolectotypes are given (if available) following the herbarium acronym.

## Results

The type status of the following names of taxa is determined, and justifications for their typification is presented.

1. *Cardamine brachycarpa* Franch., Bull. Soc. Bot. France 26: 83. 1879, nom illeg.(http://ipni.org/urn:lsid:ipni.org:names:280196-1:1.4), non Opiz, Naturalientausch 11: 411. 1826 (http://ipni.org/urn:lsid:ipni.org:names:280195-1:1.3). Described from: [JAPAN] “Insul. Nippon, prov. Etchigo, circa Niigata, secus vias humidas (R. P. Faurie)”. Lectotype (designated here): [JAPAN, Prefecture Niigata], “Nippon, Niigata, secus vias, [*U. J.*] *Faurie 23*” – P! (P00747512 [http://coldb.mnhn.fr/catalognumber/mnhn/p/p00747512]); Isolectotype – P! (P00747513 [http://coldb.mnhn.fr/catalognumber/mnhn/p/p00747513]). = ***Cardamine flexuosa*** subsp. ***debilis*** O.E.Schulz

Franchet (1879) cited only one unnumbered collection, but the only specimens at P that carry the name and exact locality information as given in the protologue are numbered [*U. J. or R. P.*] *Faurie 23*. The duplicate annotated with the species name in Franchet’s handwriting is designated here as the lectotype.

2. ***Cardamine calthifolia*** H. Lév., Bull. Géogr. Bot. 24: 281. 1914 (http://ipni.org/urn:lsid:ipni.org:names:280217-1:1.1.2.1.1.3). Described from: [CHINA, Yunnan] “Vallée de Kiao-Mé-Ti, 3000 m., mai 1913”. Lectotype [designated here (second step lectotypification after Lauener 1965: 336)]: [CHINA, Yunnan] “vallée de Kiao-mé-ti, 3000 m, *E. E. M*[*aire*] *s.n.*” – E! (E00154675 [http://data.rbge.org.uk/herb/E00154675]); Isolectotypes – E! (E00083337 [http://data.rbge.org.uk/herb/E00083337]), P! (P00747535 [http://coldb.mnhn.fr/catalognumber/mnhn/p/p00747535]).

A single collection was cited in the protologue of the name, but none of the three duplicates mentioned above carry the species name nor were they annotated by Hector Léveillé. Lauener (1965) indicated that the type is at E, but he did not specify which of the two sheets there is the type, and therefore a second-step lectotypification is provided here.

3. ***Cardamine circaeoides*** Hook.f. & Thomson, J. Proc. Linn. Soc., Bot. 5: 144. 1861 (http://ipni.org/urn:lsid:ipni.org:names:280232-1:1.1.2.1.1.3). Described from: [INDIA] “In Himalaya orientali temperata, Sikkim interiore, sylvis, alt. 5000-7000 ped. ! J. D. H. (v.v.)”. Lectotype (designated here, or perhaps holotype): [Label 1]: [INDIA] “Hab. Sikkim, Regio temp., *J. D. H*[*ooker*]”; [Label 2]: [INDIA] “295 Hab. wet wood, Gohsun, Sikkim, 5000 ft.” – K! (K000077050 [http://specimens.kew.org/herbarium/K000077050]); Doubtful isolectotypes – B! (B 10 0386925 [http://herbarium.bgbm.org/object/B100386925]), P! (P00747534 [http://coldb.mnhn.fr/catalognumber/mnhn/p/p00747534]).

The K specimen above was collected at an elevation of 5,000 ft, which is in agreement with the protologue, whereas labels of the B and P sheets indicate the elevation of 6,000‒10,000 ft. It is questionable whether the three specimens above were collected from the same area, and that is why we feel that the B and P specimens are doubtful isolectotypes.

4. ***Cardamine delavayi*** Franch., Bull. Soc. Bot. France 33: 397. 1886 (http://ipni.org/urn:lsid:ipni.org:names:280264-1:1.4). Described from: [CHINA], “Yun-nan, ad fontes prope Mo-so-yun, haud procul a Lankong; fl. fr. immat., 2 april. 1885 (Delav. n. 1838)”. Lectotype (designated here): [CHINA] [Label 1, printed] “Plantes de Chine, Province du (Yun-nan)” [Label 2, written] “… fontes prés de Mo-so-yun (Lan Kong), 2 avril 1885, *J. M. Delavay 1838*” – P! (P00279370 [http://coldb.mnhn.fr/catalognumber/mnhn/p/p00279370]); Isolectotypes – P! (P00747533 [http://coldb.mnhn.fr/catalognumber/mnhn/p/p00747533]), US! (US00324476 [http://plants.jstor.org/stable/10.5555/al.ap.specimen.us00324476]).

A single collection was cited in the original publication of this species, but the existence of two duplicates of the same collection at P and one in US annotated by Franchet calls for lectotypification, which is done here.

5. ***Cardamine elegantula*** Hook.f. & Thomson, J. Proc. Linn. Soc., Bot. 5: 146. 1861 (http://ipni.org/urn:lsid:ipni.org:names:280285-1:1.1.2.1.1.3). Described from: [BHUTAN] “In Himalaya temperata orientali, Bhotan ! alt. 6500 ped., Griffith”. Lectotype (here designated): “[BHUTAN] “Bhotan”, 1838, [*W.*] *Griffith 1360*” – K! (K000247218 [http://specimens.kew.org/herbarium/K000247218]); Isolectotypes – B! (B 10 0241392 [http://herbarium.bgbm.org/object/B100241392]), P! (P05036298 [http://coldb.mnhn.fr/catalognumber/mnhn/p/p05036298]).

Two collection numbers by William Griffith from Bhutan, *Griffith 1360* and *Griffith 1756*, are mounted on the same sheet at K. The latter collection carries no determination and is attached by two pins to the sheet of the former collection, whereas the lectotype designated here carries Hooker’s determination as “Card. elegantula Hf. & T.”, has well-developed fruits and is represented by duplicates at B and P.

6. Cardamine hirsuta var. flaccida Franch., Bull. Soc. Bot. France 33: 398. 1886. Described from: [CHINA] “Yun-nan in umbrosis et humidis ad Mo-so-yn, prope Lankong; fl. fr., 14 mart. 1885 (Delav. n. 1839)”. Lectotype (designated here): “CHINA, Yun-nan, Lieux humides et ombragés à Mo-so-yn, (Lan Kong), 14 Mart 1885, *J. M. Delavay 1839*” – P! (P00747528 [http://coldb.mnhn.fr/catalognumber/mnhn/p/p00747528]); Isolectotypes – E! (E00154697 [http://data.rbge.org.uk/herb/E00154697]), K! (K000697728 [http://specimens.kew.org/herbarium/K000697728]), P! (P00250221 [http://coldb.mnhn.fr/catalognumber/mnhn/p/p00250221], P00250222 [http://coldb.mnhn.fr/catalognumber/mnhn/p/p00250222], P00279384 [http://coldb.mnhn.fr/catalognumber/mnhn/p/p00279384], P000747522 [http://coldb.mnhn.fr/catalognumber/mnhn/p/p000747522]), US! (US00100035 [https://plants.jstor.org/stable/10.5555/al.ap.specimen.us00100035]). = ***Cardamine flexuosa*** subsp. ***debilis*** O.E.Schulz

Five duplicates of the type collection of Cardamine hirsuta var. flaccida are housed at P, and the most complete specimen with Franchet’s annotation is designated here as the lectotype.

7. *Cardamine insignis* O.E.Schulz, Bot. Jahrb. Syst. 32: 439. 1903 (http://ipni.org/urn:lsid:ipni.org:names:280398-1:1.4). Described from: “China australis: prov. Yunnan in silvis 2000 m leg. A. Henry n. 13090 (H. B. [B])”. Lectotype (designated here, or perhaps holotype): [CHINA] “Yunnan, N. Szemao, 6,000 ft., *A. Henry 13090*”. – B! (B 10 0386926 [http://herbarium.bgbm.org/object/B100386926]); Isolectotypes – E! (E00154731 [http://data.rbge.org.uk/herb/E00154731]), K! (K000697718 [http://specimens.kew.org/herbarium/K000697718]), MO! (357226 [http://www.tropicos.org/Specimen/2035994]). = ***Cardamine circaeoides*** Hook.f. & Thomson (http://ipni.org/urn:lsid:ipni.org:names:280232-1:1.1.2.1.1.3).

Schulz (1903) listed a single collection in the protologue and cited only the herbarium B. Currently there is only one specimen of this collection at B. Nevertheless, as considerable part of the Berlin herbarium was destroyed in 1943, we cannot exclude that there was originally more than one specimen of this collection at B in the past. To be on the safe side, extant specimen is treated here as “lectotype, or perhaps holotype.”

8. Cardamine macrophylla var. dentariifolia Hook.f. & T.Anderson, Fl. Brit. India 1: 139. 1872. Described from: [CHINA] “From Kumaon to Kashmir”. Lectotype (designated here): [INDIA, Uttarakhand], “Hab. Himal. Bor. Occ., Regio Temp., *T. T.* [*T. Thomson*] *s.n.*” – K! (K000397478 [http://specimens.kew.org/herbarium/K000397478]). = ***Cardamine macrophylla*** Willd. (http://ipni.org/urn:lsid:ipni.org:names:280460-1:1.1.5.1.1.1).

9. Cardamine macrophylla var. foliosa Hook.f. & T.Anderson, Fl. Brit. India 1: 139. 1872. Described from: [INDIA] “Wall. Cat. 4779.— Kumaon and Kashmir”. Lectotype (designated here): [INDIA, Uttarakhand], “Kamoon [Kumaon], Wall. Cat. 4779, *R. B.* [Robert Blinkworth] *s.n*.” – K! (K000247365 [http://specimens.kew.org/herbarium/K000247365]); Isolectotypes – B! (B 10 0241370 [http://herbarium.bgbm.org/object/B100241370], B 10 0241369 [http://herbarium.bgbm.org/object/B100241369]), P! (P00747537 [http://coldb.mnhn.fr/catalognumber/mnhn/p/p00747537]). = ***Cardamine macrophylla*** Willd. (http://ipni.org/urn:lsid:ipni.org:names:280460-1:1.1.5.1.1.1).

10. Cardamine macrophylla var. lobata Hook.f. & T.Anderson, Fl. Brit. India 1: 139. 1872. Described from: [INDIA] “Kashmir, 6000 ft.; Western Tibet, 13,000 ft.” Lectotype (designated here): [INDIA] [Label 1]: “marshy meadows, Nira Zanskar, 12,900 ft, 2 July 1849 [?]”, [Label 2]: “Hab. Himal. Bor. Occ., W. Tibet, Regio Temp., Alt. 12,900 ft, *T. T.* [*T. Thomson*] *s.n.*” – K! (K000397477 [http://specimens.kew.org/herbarium/K000397477]). = ***Cardamine macrophylla*** Willd. (http://ipni.org/urn:lsid:ipni.org:names:280460-1:1.1.5.1.1.1).

11. Cardamine macrophylla var. sikkimensis Hook.f. & T.Anderson, Fl. Brit. India 1: 139. 1872. Described from: “Inner ranges of Sikkim, 7,000-13,000 ft.”. Lectotype (designated here): [INDIA], [Label 1]: “Lachomy, 12,000 ft, 3 September 1849”, [Label 2]: “Hab. Sikkim, Regio Alp. Temp. *J. D. H.* [*Hooker*] *s.n.*” – K! (K000397479 [http://specimens.kew.org/herbarium/K000397479]); Isolectotypes – K! (K000397480 [http://specimens.kew.org/herbarium/K000397480], K000397481 [http://specimens.kew.org/herbarium/K000397481]). = ***Cardamine macrophylla*** Willd. (http://ipni.org/urn:lsid:ipni.org:names:280460-1:1.1.5.1.1.1).

Hooker and Anderson (1872) divided *Cardamine macrophylla* into four numbered varieties: “1. *dentarifolia*”, “2. *foliosa*”, “3. *lobata*”, and “4. *sikkimensis*”. The sheets at K are annotated by Hooker as “*Cardamine macrophylla*, Willd.” followed by α, β, γ, and δ. These clearly correspond to the numbers 1 to 4 cited in the above reference, respectively. The sheets best matching the descriptions of the above four varieties are designated as lectotypes.

12. Cardamine macrophylla var. moupinensis Franch., Pl. David. 2: 18. 1888. Described from: “Moupine, in umbrosis monatis. Fl. April. 1869”. Lectotype (designated here): [CHINA], [Label 1 (handwritten)]: “Moupin, Thibet oriental, lieux frais en montagne, Avril 1869”, [Label 2 (printed)] “Chine (Thibet Oriental), Province de Moupin, 1870 [sic!], *David s.n.*” – P! (P00747519 [http://coldb.mnhn.fr/catalognumber/mnhn/p/p00747519]); Isolectotype – P! (00747518 [http://coldb.mnhn.fr/catalognumber/mnhn/p/p00747518]). = ***Cardamine macrophylla*** Willd. (http://ipni.org/urn:lsid:ipni.org:names:280460-1:1.1.5.1.1.1).

The more complete specimen of the two P duplicates of the type collection is designated here as the lectotype. There is a single plant on the sheet and it seems that the year on the printed label is either a typing error or the date of accession at P.

13. ***Cardamine microzyga*** O.E.Schulz, Bot. Jahrb. Syst. 32: 545. 1903 (http://ipni.org/urn:lsid:ipni.org:names:280483-1:1.4). Described from: [CHINA] “Asia centralis ad fines prov. Tibet et West-Szechuen pr. Tachienlu 3000-4500 in leg. A. E. Pratt n. 265 ante 1890 (H. B. [B])”. Lectotype (designated here, or perhaps holotype): [CHINA] “West Sichuan and Tibetan Frontier, chiefly near Tachienlu, at 9000-13,500 ft, purchased December 1890, *A. E. Pratt 265*” – B! (B 10 0386913 [http://herbarium.bgbm.org/object/B100386913]); Isolectotype – K! (K000077046 [http://specimens.kew.org/herbarium/K0000046]).

Schulz (1903) listed a single collection in the protologue and cited only the herbarium B. Currently there is only one specimen of this collection at B. Nevertheless, as considerable part of the Berlin herbarium was destroyed in 1943, we cannot exclude that there was originally more than one specimen of this collection at B in the past. To be on the safe side, as in the case of the type of the name *Cardamine insignis* dealt with above, extant specimen is treated here as “lectotype, or perhaps holotype”. When publishing the name *Cardamine prattii*, Hemsley and Wilson (1906: 153), included in the circumscription of the taxon to which it was applied the only element mentioned in the protologue of *Cardamine microzyga* (namely collection *A. E. Pratt 265*), making the name *Cardamine prattii* illegitimate (under Art. 52 of the *Code*, McNeill et al. 2012). Therefore, *Cardamine prattii*, following the Art. 7.5 of the *Code*, should be automatically typified by the type of the name *Cardamine microzyga*.

14. ***Cardamine multijuga*** Franch., Bull. Soc. Bot. France 33: 399. 1886 (http://ipni.org/urn:lsid:ipni.org:names:280497-1:1.4). Described from: [CHINA] “Yun-nan, in aquosis ad Mo-so-yn, prope Langkong; fl. 28 jun. 1884 (Delav. n. 697)”. Lectotype (designated here): [CHINA, Yunnan] “Les fossés à Mo-so-yun (Lan Kong), 28 Juin 1884, *J. M. Delavay 697*” – P! (P00279390 [http://coldb.mnhn.fr/catalognumber/mnhn/p/p00279390]); Isolectotypes – BM!, E! (E00154552 [http://data.rbge.org.uk/herb/E00154552]), GH! (GH00112040 [http://plants.jstor.org/stable/10.5555/al.ap.specimen.gh00112040]), K! (K000697737 [http://specimens.kew.org/herbarium/K000697737], K000697738 [http://specimens.kew.org/herbarium/K000697738]), NY! (NY00468267 [http://plants.jstor.org/stable/10.5555/al.ap.specimen.ny00468267]), P! (P00747516 [http://coldb.mnhn.fr/catalognumber/mnhn/p/p00747516], P00747517 [http://coldb.mnhn.fr/catalognumber/mnhn/p/p00747517]), US! (US00100044 [http://plants.jstor.org/stable/10.5555/al.ap.specimen.us00100044], US01100650 [http://plants.jstor.org/stable/10.5555/al.ap.specimen.us01100650]). ≡ Cardamine griffithii subsp. multijuga (Franch.) O.E.Schulz.

A single collection was cited by Franchet (1886), and the best duplicate at P that he annotated is designated here as the lectotype.

15. Cardamine multijuga var. gracilis O.E.Schulz, Repert. Spec. Nov. Regni Veg. 17: 289. 1921. Described from: “China: Prov. Yunnan, im Gebiete Lichiang beim Dorfe Ugu leh keh an einem Sumpfe, ca. 2900 m ü. M. (Camillo Schneider, It. chin. 1914, no. 1862 – Mitte Juli in Blüte, Blumen weib-lila, hb. Berlin [B]”. Lectotype (designated here, or perhaps holotype): “CHINA, Yunnan: in reg. Lichiang prope pagum Ngu leh tseh, ad stagnum. 18 Jul. 1914, Alt. circiter 2900, *Camillo Schneider 1862*” – B! (B 10 0386915 [http://herbarium.bgbm.org/object/B100386915]); Isolectotypes – GH! (GH00142216 [http://plants.jstor.org/stable/10.5555/al.ap.specimen.gh00142216]), K!, US! (US00100045 [http://plants.jstor.org/stable/10.5555/al.ap.specimen.us00100045]). ≡ ***Cardamine gracilis*** (O.E.Schulz) T.Y.Cheo & R.C.Fang (http://ipni.org/urn:lsid:ipni.org:names:280343-1:1.2.1.4).

Schulz (1903) listed a single collection in the protologue and cited only the herbarium B. Currently there is only one specimen of this collection at B. Nevertheless, as considerable part of the Berlin herbarium was destroyed in 1943, we cannot exclude that there was originally more than one specimen of this collection at B in the past. To be on the safe side, as in the case of the type of the names *Cardamine insignis* and *Cardamine microzyga* dealt with above, extant specimen is treated here as “lectotype, or perhaps holotype.”

16. ***Cardamine paucifolia*** Hand.-Mazz., Symb. Sin. 7(2): 359. 1931 (http://ipni.org/urn:lsid:ipni.org:names:280343-1:1.2.1.4). Described from: [CHINA] “Y. [Yunnan] In wtp. Wäldern zwischen Dawan und Gwanyilang bei Yungbei (“Yungpeh”), Sandstein, 2400–2600 m, 3. VII. 1914 (3432)”. Lectotype (designated here): [CHINA] “Prov. Yünnan, prope urbem Yungbei, in regionis calide temperatae, silvis inter vic. Dawan et Gwangyilang, 2400–2600 m, 3. VII. 1914, *H. Freiherr v. Handel-Mazzetti 3432*” – WU! (WU024360 [http://herbarium.univie.ac.at/database/detail.php?ID=6859]); Isolectotype – W! (W1931-0001417 [http://herbarium.univie.ac.at/database/detail.php?ID=265002]).

Although Handel-Mazzetti (1931) cited a single collection of his, no indication was given as to where the type is deposited. We have been unable to find more than two sheets of the type collection, and both carry identical labels in Handel-Mazzetti’s handwriting. The WU sheet is designated as the lectotype.

17. ***Cardamine rockii*** O.E.Schulz, Notizbl. Bot. Gart. Berlin-Dahlem 9: 473. 1926 (http://ipni.org/urn:lsid:ipni.org:names:280627-1:1.4). Described from: “China: Southwestern Szechuan, Muli or Mili Kingdom, 3300–4650 m s. m., J. F. Rock, Juni 1922, n. 5585”. Lectotype (designated here): “CHINA, Muli or Muli Kingdom, southwestern Szechuan, 10,000–14,000 ft, June 1922, *J. F. Rock 5585*” – B! (B 10 0241338 [http://herbarium.bgbm.org/object/B100241338]); Isolectotypes – E (E00154543 [http://data.rbge.org.uk/herb/E00154543]), GH! (GH00312595 [http://plants.jstor.org/stable/10.5555/al.ap.specimen.gh00312595]), P! (P00747504 [http://coldb.mnhn.fr/catalognumber/mnhn/p/p00747504]), US! (US00100053 [http://plants.jstor.org/stable/10.5555/al.ap.specimen.us00100053]).

Schulz (1926) listed a single collection in his description of the species, and the duplicate at B is designated as the lectotype because it was studied and annotated by him.

18. *Cardamine scoriarum* W.W.Sm., Notes Roy. Bot. Gard. Edinburgh 11: 203. 1919 (http://ipni.org/urn:lsid:ipni.org:names:280650-1:1.3). Described from: “China: - Yunnan; flank of volcanic mountain to north-west of Teng-yueh. Lat. 25°10'N, Alt. 7000 ft. Moist shady situations in thickets. June 1912, G. Forrest. No. 8201. Yunnan; divide between the Shweli and Teng-yueh valleys. Lat. 25°N. Alt. 6000–7000 ft. Moist shady situations on the margins of thickets. May 1912. G. Forrest. No. 7947”. Lectotype (designated here): [CHINA] “Yunnan, West China, flank of volcanic mountain north-west of Tenguyeh, 25°70¢N, 7000 ft., June 1912, *G. Forrest 8201*” – E! (E00154541 [http://data.rbge.org.uk/herb/E00154541]); Isolectotype – K! (000697740 [http://specimens.kew.org/herbarium/K000697740]). º *Cochlearia scoriarum* (W.W.Sm.) Hand.-Mazz. (http://ipni.org/urn:lsid:ipni.org:names:281500-1:1.5.2.1) = ***Cardamine fragariifolia*** O.E.Schulz (http://ipni.org/urn:lsid:ipni.org:names:280320-1:1.4).

Smith (1919) cited two collections by George Forrest, those of *Forrest 7947* [E! (E00117483 [http://data.rbge.org.uk/herb/E00117483]), K! (K000697741 [http://specimens.kew.org/herbarium/K000697741])] were examined by us as well. The above lectotype collection is better than the other, and the E sheet was annotated in Smith’s handwriting as “type”, and it carries the original printed label with Forrest’s handwritten locality data. By contrast, label of the K duplicate was typed at some later time.

19. ***Cardamine simplex*** Hand.-Mazz., Symb. Sin. 7(2): 361. 1931 (http://ipni.org/urn:lsid:ipni.org:names:280661-1:1.3.2.1). Described from: “NW-Y. [Yunnan], Sumpfwiesen der tp. St. ober Ganhaidse bei Lidjiang, Sandstein, 3200 m, 22. VII. 1914 (4310). Offene Stellen an Bächen am Osthange des Beimaschan zwischen Djinscha-djiang und Mekong, 28°12’, 3300 m, VI. 1917 (Forrest 13840)”. Lectotype (designated here): [CHINA] “Prov. Yünnan bor.-occid.: Supra vicum Ganhaidse ad urbem Lidjiang (“Likiang”), in regionis temperatae pratis paludosis, alt. s. m. ca. 3200 m, 22. VII. 1914, *H. Freiherr von Handel-Mazzetti 4310*” – WU! (WU0024361 [http://herbarium.univie.ac.at/database/detail.php?ID=6858]); Isolectotypes – E! (E00154534 [http://data.rbge.org.uk/herb/E00154534]), GH! (GH00112038 [http://plants.jstor.org/stable/10.5555/al.ap.specimen.gh00112038]), NAS!, W! (1931-0011416 [http://herbarium.univie.ac.at/database/detail.php?ID=264999]).

Two collections were cited by Handel-Mazzetti (1931) in the original description of the species, and all specimens/duplicates studied were annotated by the author/collector of the species/specimens in his handwriting as “*Cardamine simplex* Hand.-Mazt., sp. nova.” Specimen of his own collection is designated here as the lectotype. Annotated specimen of the collection *Forrest 13840* was found at W! (W1929-0013606 [http://herbarium.univie.ac.at/database/detail.php?ID=216904]).

20. ***Cardamine tangutorum*** O.E.Schulz, Bot. Jahrb. Syst. 32: 360. 1903 (http://ipni.org/urn:lsid:ipni.org:names:280688-1:1.4). Described from: “China: Prov. Kansu in terra Tangutorum leg. N. M. Przewalski 1872 (H. B. Boiss. [G], H. P. Ac. [LE]), 1873 (H. B. [B]), 1880 (H. P. Ac. [LE]), prov. Kansu orient, leg. G. N. Potanin 1885 (H. B. [B]), occid. leg. idem (H. P. [LE]); prov. Schensi sept.: Miao Wang-san pr. Paoki-scen leg. J. Giraldi 1899 No. 3379, in alto monte Thae-pei-san leg. idem No. 3378 (H.B. [B]); prov. Schansi leg. Potanin 1884 (H.P.Ac. [LE]); Flora Pekinensis, in m. Siao-Wu-Tai-shan 1660–2330 m leg. O. v. Möllendorff 1879 (H. B. [B]); prov. Szetschuan sept. leg. idem 1885 (H.P.Ac. [LE]), ad Tsakulao leg. V. Rosthorn 1891 No. 2583 (H. B. [B])”. Lectotype (designated here): “CHINA occidentalis, Terra Tangutorum, (prov. Kansu [Gansu Province]), 1873, *N. M. Przewalski s.n.*” – B! (B 10 0241335 [http://herbarium.bgbm.org/object/B100241335]); Isolectotypes – K! (K000697742 [http://specimens.kew.org/herbarium/K000697742]), LE!.

Schulz (1903) cited 10 syntype collections under this species and gave the institutional abbreviations where the duplicates are housed. The lectotype was annotated by him, and we have examined its duplicates in other herbaria (K, LE).

21. ***Cardamine trifoliolata*** Hook.f. & Thomson, J. Proc. Linn. Soc., Bot. 5: 145. 1861 (http://ipni.org/urn:lsid:ipni.org:names:280717-1:1.1.2.1.1.3). Described from: “In Himalaya orientali, reg. temp., Bhotan ! Griffith.” Lectotype (designated here): [BHUTAN] “Bootan, [1838], *W. Griffith 1757*” – K! (K000247214 [http://specimens.kew.org/herbarium/K000247214]).

Two of Griffith’s collection numbers from Bhutan, *Griffith 1757* and *1359*, are mounted on the same sheet. The former collection was cut off and mounted on the sheet of the other, and it is the one with Hooker’s annotation as “*Cardamine trifoliolata*, Hf & T” that we designate herein as the lectotype.

22. *Cardamine violifolia* O.E.Schulz, Bot. Jahrb. Syst. 32: 440. 1903 (http://ipni.org/urn:lsid:ipni.org:names:280743-1:1.4). Described from: “China centralis: prov. Hupeh pr. Ichang leg. A. Henry 10. 1887 n. 3298 (H. B. [B], H. B. Boiss. [G], H. C. [GH])”. Lectotype (designated here): [CHINA] “Central China, prov. Hupeh [Hubei], *Ichang and intermediate neighborhood* [more detailed information in italic is given on the other specimens of this collection than lectotype], 1885-1888, *A. Henry 3298*” – B! (B 10 0386924 [http://herbarium.bgbm.org/object/B100386924], except middle plant on the sheet); Isolectotypes – BM! (BM000587602 [http://plants.jstor.org/stable/10.5555/al.ap.specimen.bm000587602]), E! (E00154597 [http://data.rbge.org.uk/herb/E00154597]), G (ex Herbarium Barbey-Boissier)!, GH! (GH00112032 [http://plants.jstor.org/stable/10.5555/al.ap.specimen.gh00112032], except the plant on the right; GH00112031 [http://plants.jstor.org/stable/10.5555/al.ap.specimen.gh00112031]), K!, P! (P05036305 [http://coldb.mnhn.fr/catalognumber/mnhn/p/p05036305]), US! (US00100059 [http://plants.jstor.org/stable/10.5555/al.ap.specimen.us00100059]). = ***Cardamine circaeoides*** Hook.f. & Thomson (http://ipni.org/urn:lsid:ipni.org:names:280232-1:1.1.2.1.1.3).

Schulz (1903) listed one collection and cited the duplicates at B, G, and GH. We have examined all four sheets that he cited, and any of them could have served as the lectotype, but Schulz’s institutional affiliation was the reason to designate the B duplicate as the lectotype.

23. Cardamine violifolia var. diversifolia O.E.Schulz, Bot. Jahrb. Syst. 32: 440. 1903. Described from: “Saepe cum specie typica.” Lectotype (designated here): [CHINA] “Central China, prov. Hupeh [Hubei], *Ichang and intermediate neighborhood* [more detailed information in italic is given on other specimens of this collection than lectotype], 1885-1888, *A. Henry 3298*” – B! (same as above) (B 10 0386924 [http://herbarium.bgbm.org/object/B100386924], middle plant on the sheet); isolectotype – GH! (GH00112032 [http://plants.jstor.org/stable/10.5555/al.ap.specimen.gh00112032], plant on the right). = ***Cardamine circaeoides*** Hook.f. & Thomson (http://ipni.org/urn:lsid:ipni.org:names:280232-1:1.1.2.1.1.3).

According to his annotations on the herbarium sheets, both the B and GH sheets were considerd by Schulz (1903) as a mixed collection of the type variety and var. *diversifolia*. The latter variety was said to differ by having slightly lobed vs. unlobed leaves, though this variation is quite frequently encountered in almost every population of the species and, therefore, we considered it trivial and not worth of taxonomic classification.

24. ***Cardamine yunnanensis*** Franch., Bull. Soc. Bot. France 33: 398. 1886 (http://ipni.org/urn:lsid:ipni.org:names:280759-1:1.4). Described from: “[CHINA] Yun-nan, in silvis ad Ta-long-tan, prope Tapin-tze, alt. 1800 m.; fl., fr. 26 jul. 1885 (Delav. n. 1843)”. Lectotype (designated here): [Label 1, printed]: “Plantes de Chine (Province du Yun-nan)”, [Label 2, handwritten] “Les bois de Ta-long-tan, près de Ta pint tze, à 1800m d’altitude, 26 juillet 1885, *J. M. Delavay 1843*” – P! (P00747493 [http://coldb.mnhn.fr/catalognumber/mnhn/p/p00747493]); Isolectotypes – BM!, E! (E00154592 [http://data.rbge.org.uk/herb/E00154592]), F! (F0092968F [http://cornelia.fieldmuseum.org/285/783/V0092968F.jpg]), NY! (NY00468268 [http://plants.jstor.org/stable/10.5555/al.ap.specimen.ny00468268]), P! (P00747494 [http://coldb.mnhn.fr/catalognumber/mnhn/p/p00747494], P00747495 [http://coldb.mnhn.fr/catalognumber/mnhn/p/p0074495]), US! (US00100060 [http://plants.jstor.org/stable/10.5555/al.ap.specimen.us00100060], US00503998 [http://plants.jstor.org/stable/10.5555/al.ap.specimen.us00503998]).

A single collection was cited in the original publication. Of the three duplicates of the type collection at P, two have Delavay’s handwritten label and Franchet’s annotation. The most complete one is designated here as the lectotype.

25. *Dentaria repens* Franch., Bull. Soc. Bot. France 32: 5. 1885 (http://ipni.org/urn:lsid:ipni.org:names:281996-1:1.5). Described from: [CHINA] “Plantes du Yun-nan. In faucibus Han-tchang-kiou, secus viam e Tali ad Ho-kin ducentem; 27 maj. 1884; n°65”. Lectotype (designated here): [CHINA, Yunnan] “Gorges de San tchang kiou. Ho Kin, 22 May 1884, *J. M. Delavay s.n.*” – P! (P00747505 [http://coldb.mnhn.fr/catalognumber/mnhn/p/p00747505]); Isolectotype – P! (P00747506 [http://coldb.mnhn.fr/catalognumber/mnhn/p/p00747506]). ≡ ***Cardamine repens*** (Franch.) Diels (http://ipni.org/urn:lsid:ipni.org:names:280619-1:1.3). ≡ Cardamine tenuifolia var. repens (Franch.) Franch. º *Loxostemon repens* (Franch.) Hand.-Mazz. (http://ipni.org/urn:lsid:ipni.org:names:286642-1:1.3.2.1.1.1).

Franchet (1885) dealt with Delavay’s collections and listed only collection number 65 under the species. The only material at P with *Delavay 65* is a unicate of Saxifraga diversifolia var. hematophylla Franch. (P00136633). However, two sheets at P with the exact locality data (Han tchang-kiou, Ho-Kin) but without any collection number carry Franchet’s annotation as “Cardamine tenuifolia Turcz. var. repens Franch.,” and both were collected on May 22 (not 27, as in the protologue) of 1884. The sheet with the original hand-written label by Delavay, which carries the name “*Dentaria repens* Franch.”, is designated herein as the lectotype. There are no other Delavay specimens of *Cardamine* or *Dentaria* at P that carry the above information and, therefore, it is safe to conclude that Franchet (1885) made mistakes in collection day and number.

26. *Erysimum violaceum* D.Don, Prodr. Fl. Nepal.: 202. 1825 (http://ipni.org/urn:lsid:ipni.org:names:284251-1:1.2.2.1.1.1). Described from: [NEPAL] “Hab. in Gosaingsthan. *Wallich*.” Lectotype (designated here): [NEPAL] “Gosain Than, *N. Wallich 4782*” – K! (K000247213 [http://specimens.kew.org/herbarium/K000247213]); Isolectotypes – BM! (BM000521637 [http://plants.jstor.org/stable/10.5555/al.ap.specimen.bm000521637]), B! (B 10 0241325 [http://herbarium.bgbm.org/object/B100241325]), E! (E00154660 [http://data.rbge.org.uk/herb/E00154660]), GZU! (GZU000276995 [http://plants.jstor.org/stable/10.5555/al.ap.specimen.gzu000276995]). ≡ ***Cardamine violacea*** (D.Don) Wall. ex Hook.f. & Thomson (http://ipni.org/urn:lsid:ipni.org:names:280742-1:1.2.1.1.2.1.1.1).

According to Stafleu and Cowan (1988: 37‒38), Nathaniel Wallich’s complete set of Himalayan plants is currently deposited at K, and David Don’s types in the “*Prodromus florae nepalensis*” (Don 1825) that were based on Wallich’s collections were sent to him by Lambert and partially housed at BM. The duplicate at K is the most complete of all that we examined, and it is designated herein as the lectotype.

27. *Loxostemon delavayi* Franch., Bull. Soc. Bot. France 33: 400. 1886 (http://ipni.org/urn:lsid:ipni.org:names:286639-1:1.4), non *Cardamine delavayi* Franch., Bull. Soc. Bot. France 33: 397. 1886 (http://ipni.org/urn:lsid:ipni.org:names:280264-1:1.4). Described from: [CHINA] “Yun-nan ad juga nivalia Li-kiang post nives deliquescentens florens; fl. 9 jul. 1884 (Delavay)”. Lectotype (designated here): [CHINA] [Label 1, printed] “Yunnan”, [Labe1 2, written] “parmi les pierres après la fonte des neiges au Glacier Li Kiang, 9 iuillet 1884, *J. M. Delavay 35*” – P! (P00279378 [http://coldb.mnhn.fr/catalognumber/mnhn/p/p00279378]); Isolectotypes – P! (P00747524 [http://coldb.mnhn.fr/catalognumber/mnhn/p/p00747524], P00747525 [http://coldb.mnhn.fr/catalognumber/mnhn/p/p00747525]). ≡ ***Cardamine franchetiana*** Diels (http://ipni.org/urn:lsid:ipni.org:names:280322-1:1.3).

No collection number was given by Franchet (1886) in the original publication of *Loxostemon delavayi*, and the only collection carrying Franchet’s annotation of the species and collected on the exact date cited in that publication is *Delavay 35*. That number was cited by Diels (1912) in renaming the species during transferring it to *Cardamine* to avoid the creation of a later homonym of *Cardamine delavayi*, which is an entirely different species. The lectotype is the more complete of the three sheets at P and has five plants mounted on the sheet, together with author’s drawing of details of flowers and fruit.

28. *Loxostemon pulchellus* Hook.f. & Thomson, J. Proc. Linn. Soc., Bot. 5: 147. 1861 (http://ipni.org/urn:lsid:ipni.org:names:286641-1:1.1.2.1.1.3). Described from: [CHINA] “In Himalaya orientali temperata; Sikkim graminosis humidis! alt. 10000-13000 ped., J. D. H. (fl. Jun.) (v.v.)”. Lectotype: [INDIA] [Label 1, printed] “Hab. Sikkim, Regio Alp” [Label 2, writeen] “Streams, Lachen, 12,000 ft, 9 June 1849, *J. D. Hooker s.n*.” – K! (K000397471 [http://specimens.kew.org/herbarium/K000397471]); Possible isolectotypes – B! (B 10 0241277 [http://herbarium.bgbm.org/object/B100241277]), P! (P00747596 [http://coldb.mnhn.fr/catalognumber/mnhn/p/p00747596], P00747597 [http://coldb.mnhn.fr/catalognumber/mnhn/p/p00747597], P00747598 [http://coldb.mnhn.fr/catalognumber/mnhn/p/p00747598]). ≡ ***Cardamine pulchella*** (Hook.f. & Thomson) Al-Shehbaz & G.Yang (http://ipni.org/urn:lsid:ipni.org:names:1002420-1:1.1.2.1.1.2).

Six collections of Joseph Dalton Hooker from Sikkim are mounted on two herbarium sheets at K, and they vary in the elevations and dates of collection. The single collection in which the habitat was given is designate herein as the lectotype. The Sikkim duplicates at B and P do not carry the exact elevations and collection dates of the lectotype and, therefore, we are uncertain if they are part of the same collection.
